# Editorial: Innate and adaptive immunity against tuberculosis infection: diagnostics, vaccines, and therapeutics

**DOI:** 10.3389/fimmu.2024.1366976

**Published:** 2024-01-23

**Authors:** Zhidong Hu, Lanbo Shi, Jianping Xie, Xiao-Yong Fan

**Affiliations:** ^1^ Shanghai Public Health Clinical Center & Shanghai Institute of Infectious Disease and Biosecurity, Fudan University, Shanghai, China; ^2^ Public Health Research Institute, New Jersey Medical School, Rutgers Biomedical and Health Sciences, Rutgers, The State University of New Jersey, Newark, NJ, United States; ^3^ Chongqing Municipal Key Laboratory of Karst Environment, School of Life Sciences, Southwest University, Chongqing, China

**Keywords:** tuberculosis, innate immunity, adaptive immunity, diagnostics, therapeutics, vaccines

Tuberculosis (TB) remains one of the major causes of infectious disease mortality to this day. According to the latest “Global Tuberculosis Report” released by WHO, there were 10.6 million new cases and 1.3 million deaths in 2022 (1). Thus, TB remains the world’s most lethal infectious disease, only being surpassed by COVID-19 during the 2019-2021 pandemic.

TB is caused by the acid-fast bacillus *Mycobacterium tuberculosis* (*Mtb*), which was identified by Robert Koch in 1882. One of the fundamental pillars to reduce the spread of *Mtb* infection is accurate and rapid diagnostics. The current TB diagnostics include culture, smear, GeneXpert MTB/RIF (Xpert), interferon-gamma release assays (IGRAs), imaging examination, etc. However, the traditional detection of growth in bacterial cultures is time-consuming; the sensitivity of acid-fast staining-based smear diagnostic is low; Xpert is expensive and impractical for widespread clinical use in developing countries although it is rapid and sensitive; IGRAs cannot distinguish between asymptomatic latent TB infection and active TB disease; and the specificity of imaging examination is low (2–4). Thus, the diagnosis of TB remains challenging. In this editorial, firstly, we introduce a Research Topic that include a number of studies that investigated novel diagnostic methods in the diagnosis of TB and several review papers that focused on different topics and indicated directions for future research.

The antigens ESAT-6/CFP10 (EC), which are *Mtb*-specific proteins and are absent in BCG strains, have been widely used as *Mtb*-specific stimulators in IGRA diagnosis. In a prospective cohort study, Yuan et al. enrolled 357 patients to evaluate the sensitivity and specificity of this EC skin test, which was performed by intradermal injection of recombinant EC proteins. Their data showed that the sensitivity and specificity of the EC skin test for patients were 71.52% and 65.45%, based on the clinical reference standards. Phat et al. investigated the expression of lipid-related genes during anti-TB chemotherapy through a targeted and knowledge-based approach, to evaluate the potential use of lipid-related genes as prognostic biomarkers of treatment responses. Their data showed that transcriptomic signatures of lipid-related genes were associated with the immune responses, and might be useful for treatment prognosis and TB diagnosis. Ashenafi et al. explored the peripheral inflammatory immune profiles of different TB patient sub-groups based on disease severity, anemia, and radiological performance of lung diseases. The Bio-Plex Magpix multiplex assays were used to detect cytokines in plasma and cell culture supernatants from whole blood stimulation with the EC antigens that were used in the IGRA assay. Their data suggest that inflammatory immune profiles were related to the clinical disease severity, and the top-ranked inflammatory mediators might be used as biomarkers of TB disease severity and treatment monitoring.

Gumbo et al. evaluated the performance of currently available immunological assays, including QFT, tuberculin skin test (TST), and Xpert Ultra, on detecting *M. bovis* infection in leopards (*Panthera pardus*), an African big cat population. Their preliminary results showed that TST might be a suitable tool to identify *M. bovis*-infected leopards, and the Xpert Ultra provided rapid detection of infected leopards. Corrêa et al. selected a set of candidate genes previously described to be associated with pulmonary TB and evaluated their transcriptional signatures in clinical samples from a Brazilian cohort of pleural TB patients. As a result, three genes (*CARD17*, *GBP2*, and *C1QB*) showed promise in discriminating pleural TB from other causes of exudative pleural effusion.

Wang et al. summarized the current studies demonstrating the functions of exosomes, including miRNA, circRNA, and protein, in *Mtb* infection, and discussed the potential values of exosomes as biomarkers to be used in TB diagnosis and treatment monitoring. The potential usage of exosomes in blood-based diagnostics of TB is anticipated but will need to be optimized in future studies. Another review written by Huang et al. systematically reviewed the development and clinical evaluation of proposed CRISPR-based technology in TB diagnostics, and they gave constructive suggestions on improving sample pretreatment, method development, and clinical validation of the current assays to enhance their development and translation. The booming development of CRISPR-based technology has the potential to overcome the weaknesses of current TB diagnostics and simplify sample collection by using blood or fecal specimens to give accurate results.

As the only licensed TB vaccine, immunization of *M. bovis* Bacille Calmette-Guérin (BCG) in infancy offers protection against the aggressive childhood forms of the disease including meningeal and miliary TB (5). However, its protective efficacy against TB diseases ranges from 0% to 80% in adolescents and adults (6), leading to increased morbidity among these populations (7). Thus, the lack of an optimal TB vaccine is believed to be one of the crucial barriers to global TB control, and a more effective TB vaccine is required, particularly in adolescents and adults (8). In this editorial, secondly, we summarized several studies/reviews that investigated/summarized next-generation vaccine design against TB, which aims to accelerate TB vaccine research.

Kim et al. systematically described five hurdles they think should be overcome to develop more effective TB vaccines, and then discussed the current knowledge gaps between preclinical and clinical studies regarding peripheral versus tissue-resident immune responses, different individual conditions, and correlates of protection (COP) findings. Finally, they proposed that the recent discoveries on TB risk/susceptibility-related factors could be utilized as novel biomarkers or COP, for better evaluating/predicting vaccine-induced protection against *Mtb* infection, which will facilitate the novel TB vaccine development process. Zhang et al. summarized recent progress in subunit protein vaccines against TB research. The development of bioinformatics and structural biology techniques has greatly facilitated the screening and optimization of protective *Mtb* antigens during the past decades, and the design of multistage subunit vaccines containing multiple antigens in different growth stages of *Mtb* will somewhat overcome the shorting comings of limited antigen numbers in subunit vaccines.

The development of novel adjuvants will further improve the immunogenicity of subunit vaccines. The family of proteins Pro-Glu motif-containing (PE) and Pro-Pro-Glu motif-containing (PPE) account for as much as 10% of the genome of *Mtb*, which has been found to play crucial roles in pathogenesis and persistent infection. Guo et al. reviewed the immunological regulation effects of PE/PPE proteins and the development of PE/PPE family proteins-based novel TB vaccines, including protein-based, virus vector-based, and recombinant BCG vaccines. The current studies suggest that the PE/PPE family of proteins is a highly active and promising recent area research of for TB vaccines. García-Bengoa et al. explored the immunogenicity and protection efficacy against *Mtb* infection of three PE/PPE proteins, PE18, PE31, and PPE26. As a result, all three proteins are immunoreactive in TB patients, IGRA-positive latent infected close contacts, and BCG-vaccinated healthy controls. The three antigens also induced antigen-specific T-cell immune responses and antibody responses in PBMCs and bronchoalveolar lavage in murine models. However, these antigens did not show protection in a low-dose murine aerosol *Mtb* infection model. Marques-Neto et al. evaluated a recombinant BCG vaccine encoding LTAK63 (an adjuvant that genetically detoxified a derivative of the subunit A from *E. coli* heat-labile toxin) in murine models. Their data showed that this novel vaccine induced robust and long-term Th1/Th17 T-cell immune response in the draining lymph nodes and the lungs, which was responsible for the increased protection post-*Mtb* infection six months after immunization.

As an intracellular bacterium, *Mtb* colonizes inside cells, thus, the host inflammatory and adaptive cellular immune responses, as well as the basic cellular physiologic mechanisms play important roles in the establishment of *Mtb* infection and progression of TB diseases (9, 10). In this editorial, thirdly, we summarized several studies that focus on deciphering host immune profiles against *Mtb* infection which might facilitate the anti-TB host-directed therapeutics research.

Tuberculous pleural effusion (TPE) is characterized by an influx of immune cells to the pleural space and was regarded as an appropriate platform for dissecting complex tissue responses against *Mtb* infection. Yang et al. employed a single-cell RNA sequencing study using ten pleural fluid samples from six patients with TPE and four without TPE including two from patients with transudative pleural effusion and two from patients with malignant pleural effusion, and a distinct local immune response was observed. During the process of *Mtb* infection, the levels of major acute phase protein serum amyloid A (SAA), increase up to 100-fold in the pleural fluids. However, the stimulating effects of SAA on macrophages that have not yet been in contact with mycobacteria have not been discovered yet. Kawka et al. evaluated the functional responses of human monocyte-derived macrophages under elevated SAA conditions using RNA-seq assays. Their data suggest that the presence of SAA during *Mtb* infection elevates the innate (MHC-I engagement of natural killer cells) and adaptive (MHC-I through peptides presented to cytotoxic T cells and MHC-II) immune responses induced by macrophages. Kumar et al. found that the incidence of bovine TB reactors is higher in crossbred than indigenous cattle in India, which was associated with several innate immunological factors. Their data provided a reason for adopting an appropriate crossbreeding policy that balances production and disease-resistance traits for sustainable livestock farming.

Taken together, these manuscripts published within this Research Topic provide novel information on the innate and adaptive immunity against TB infection. With more advanced knowledge, we are hopeful that more accurate diagnostics and more effective vaccines/therapeutics against *Mtb* infection will be achieved in the near future.

## Author contributions

ZH: Conceptualization, Writing – original draft, Writing – review & editing. LS: Conceptualization, Writing – review & editing. JX: Conceptualization, Writing – review & editing. X-YF: Conceptualization, Writing – review & editing.
